# La maladie du poumon des éleveurs d´oiseaux chez un enfant: à propos d´un cas rare et inhabituel

**DOI:** 10.11604/pamj.2020.37.189.26003

**Published:** 2020-10-29

**Authors:** Abderrahmane Jallouli, Karima El Fakiri, Noureddine Rada, Ghizlane Draiss, Mohammed Bouskraoui

**Affiliations:** 1 Unité de Pneumopédiatrie, Service de Pédiatrie A, Centre Hospitalier Universitaire Mohammed VI de Marrakech, Marrakech, Maroc,; 2 Faculté de Médecine et de Pharmacie, Université Cadi Ayyad de Marrakech, Marrakech, Maroc

**Keywords:** Maladie du poumon des éleveurs d’oiseaux, pneumopathie interstitielle, pneumopathie d’hypersensibilité, enfant, Bird fancier's disease, interstitial lung disease, hypersensitivity pneumonitis, child

## Abstract

La pathologie du poumon des éleveurs d´oiseaux fait partie des pneumopathies d´hypersensibilité, elle est secondaire à une réaction immuno-allergique aux antigènes aviaires. Cette entité demeure rare chez l´enfant. Elle constitue les deux tiers des pneumopathies d´hypersensibilité. Le diagnostic de cette maladie n´est pas si aisé. Il repose sur un faisceau d´arguments. Nous rapportons une observation singulière d´une fille de 7 ans, ayant comme antécédent un terrain d´atopie familiale, traitée initialement comme asthme devant des épisodes de dyspnée sifflante et une toux sans amélioration. La symptomatologie s´est aggravée par l´installation d´une dyspnée au repos, compliquée d´une détresse respiratoire cyanosante dans un contexte d´altération de l´état général. L´examen clinique a retrouvé un enfant ayant un retard pondéral, une cyanose péribuccale avec un hippocratisme digital. L´auscultation pulmonaire a objectivé des râles crépitants bilatéraux. La radiographie du thorax a mis en évidence un syndrome interstitiel bilatéral. La tomodensitométrie (TDM) thoracique a montré un aspect en verre dépoli diffus. Le bilan biologique a révélé une hyperéosinophilie avec une hyper-IgE, et a écarté une tuberculose, une mucoviscidose, un déficit immunitaire. La reprise de l´interrogatoire a montré un contact avec les oiseaux. Les sérologies de la maladie du poumon des éleveurs d´oiseaux étaient positives. La patiente est mise sous corticothérapie inhalée avec éviction de l´expositions aux oiseaux. Après un recul de 2 mois, l´évolution était favorable. Etant donné que les signes de la maladie du poumon des éleveurs d´oiseaux sont non spécifiques, elle doit être évoquée devant toute symptomatologie respiratoire avec la notion de contact aux antigènes aviaires.

## Abstract (en)

Bird fancier's disease falls within the spectrum of hypersensitivity pneumonitis secondary to immuno-allergic reaction to avian antigens. This occurs only rarely in children. It is found in two-thirds of patients with hypersensitivity pneumonitis. Diagnosis is not so easy. It is based on a body of clinical evidence. We here report the peculiar case of a 7-year-old girl with a family history of atopic disease initially treated as asthma based on the presence of wheezing and dyspnea and cough without improvement. The patient had worsening of symptoms such as dyspnea at rest complicated by cyanosis in respiratory distress. All of this took place in a context of alteration of general state. Clinical examination showed growth retardation, perioral cyanosis with digital hippocratism. Lung auscultation revealed bilateral crackling sounds. Chest X-ray objectified bilateral interstitial syndrome. Chest computed tomography (CT) scan showed diffuse ground-glass opacities. Laboratory tests revealed hypereosinophilia with hyper-IgE and excluded tuberculosis, cystic fibrosis, immune deficiency. In a second stage interview contact with birds was reported. Serological tests for bird fancier's disease were positive. The patient received inhaled corticosteroids associated with avoidance of exposure to birds. After a follow-up of 2 months, outcome was favorable. Given that the signs of bird fancier's disease are non-specific, this should be suspected in patients with respiratory symptoms associated with exposure to avian antigens.

## Introduction

La maladie du poumon des éleveurs d´oiseaux est une pathologie très rare chez l´enfant, elle est secondaire à une réaction immuno-allergique de l´organisme face à des antigènes aviaires. C´est l´étiologie la plus fréquente des pneumopathies d´hypersensibilité (PHS); constituant les deux tiers. La prévalence des PHS chez la population pédiatrique est estimée à 4 cas par million d´enfants [1]. Le diagnostic de la maladie du poumon des éleveurs d´oiseaux repose sur un faisceau d´arguments anamnestiques, cliniques et paracliniques vu les signes non spécifiques de cette entité, qui peuvent mimer d´autres pathologies. Nous rapportons après un consentement libre et éclairé des parents, une observation d´une fille de 7 ans, présentant la maladie du poumon des éleveurs d´oiseaux, diagnostiquée et traitée initialement comme asthme.

## Patient et observation

Il s´agit d´une fille de 7 ans, issue d´un mariage non consanguin, résidente dans un milieu urbain. Admise au service des urgences pédiatriques dans un tableau de détresse respiratoire cyanosante. A l´interrogatoire, l´enfant était bien vacciné selon le programme national d´immunisation, n´ayant pas de contage tuberculeux récent connu, et suivie pour un asthme non contrôlé depuis un an retenu devant des épisodes de dyspnée sifflante, une toux chronique et la notion d´atopie familiale chez la mère et le frère.

Le début de la symptomatologie remonte à l´âge de 6 ans par l´installation d´une toux sèche associée à une dyspnée sifflante à l´effort puis au repos évoluant progressivement dans un contexte d´altération de l´état général. Devant cette symptomatologie et le terrain d´atopie familiale, le diagnostic d´asthme a été retenu, et la fille a été traitée par corticoïde inhalé puis l´association corticoïde inhalé et bronchodilatateur à longue durée d´action avec une légère amélioration clinique selon la maman. La symptomatologie récente remonte à 3 mois par la récidive de la dyspnée au repos et la toux sèche, s´aggravant par l´installation d´une détresse respiratoire cyanosante sans autres signes associées notamment pas d´hémoptysies ni de troubles digestifs. Le tout évoluant dans un contexte de sueurs nocturnes et d´altération de l´état général. A l´admission, la patiente était polypnéique à 30 cycles par minutes, normocarde à 97 battements par minutes, avec une cyanose péribuccale, sans signes de lutte respiratoire. La saturation en O2 était à 92% à l´air libre et 98% sous lunettes d´oxygène avec un débit de 2 litres/minute. La température était à 37°C, le poids à 16 kg (-3DS), la taille à 121cm (M) avec un hippocratisme digital. L´auscultation objectivait des râles crépitants bilatéraux diffus. L´examen cardio-vasculaire était sans anomalies. La bandelette urinaire était normale.

**Figure 1:**
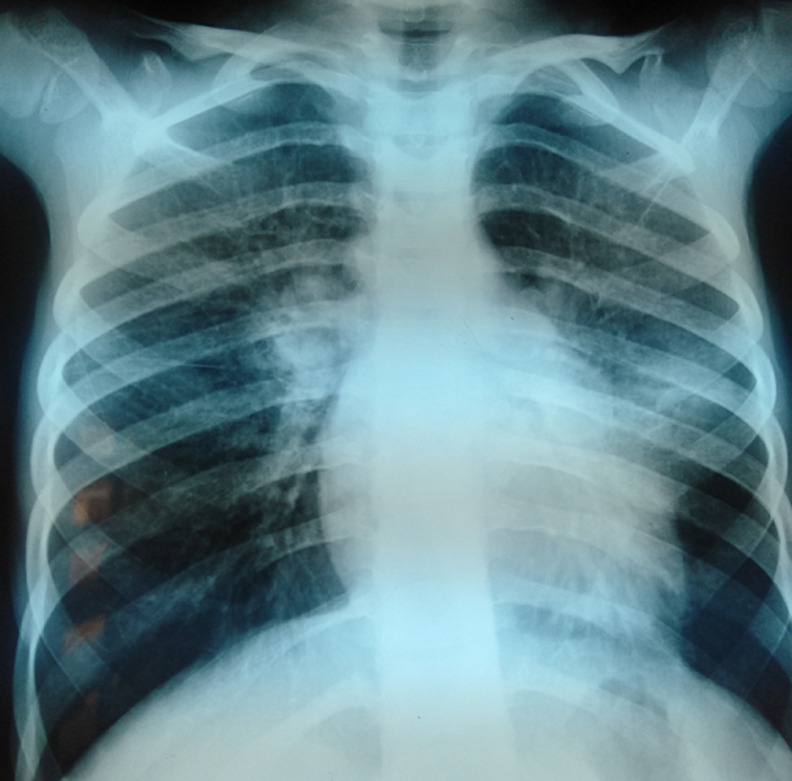
syndrome interstitiel bilatéral à la radiographie du thorax

**Figure 2:**
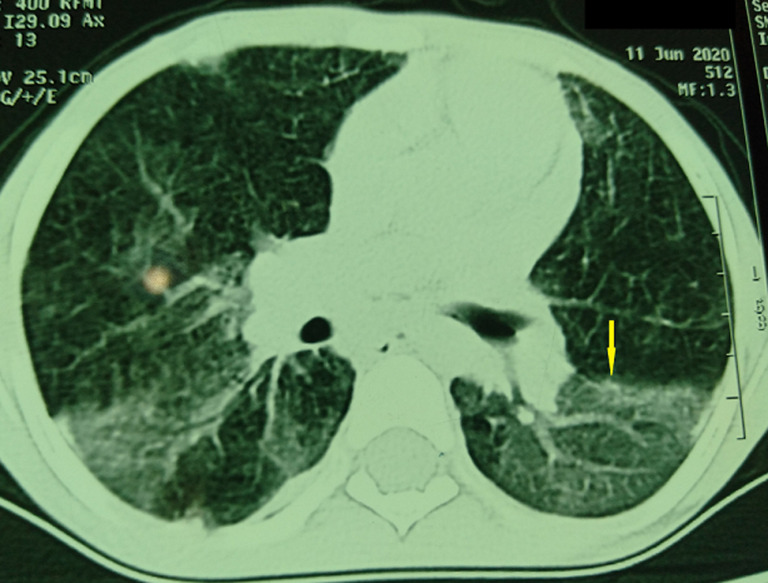
aspect en verre dépoli et en mosaïque des deux hémichamps pulmonaires

La radiographie thoracique de face a objectivé un syndrome interstitiel diffus avec une distension thoracique faisant évoquer une tuberculose, une pneumopathie interstitielle diffuse ou un déficit immunitaire (Figure 1). La TDM thoracique a montré un aspect en verre dépoli et en mosaïque du parenchyme pulmonaire intéressant les deux poumons de répartition inhomogène épargnant les régions basales, une discrète dilatation de bronches de type cylindrique du culmen, associés à un pneumomédiastin et un emphysème sous cutané (Figure 2). L´exploration fonctionnelle respiratoire a mis en évidence un syndrome restrictif sévère. La RT-PCR du SARS-CoV-2 a été négative. L´échocardiographie était normale avec une bonne fonction systolique du VG et sans signes d´HTAP.

Le bilan biologique a montré une hyperéosinophilie à 910 éléments/mm^3^. Trois BK expectorations étaient négatives. Le bilan du déficit immunitaire a objectivé une hyper IgE à 174.5 UI/ml, le test rapide du VIH était négatif. Les sérologies aspergillaires ainsi que l´examen parasitologique des selles sont revenus négatifs. Le test de la sueur était également normal à 35 mmol/l éliminant une mucoviscidose. Durant son hospitalisation, la patiente a été mise sous amoxicilline acide clavulanique à dose de 100 mg/kg/j pendant dix jours, avec une amélioration clinique et une prise de poids d´un kilogramme. A sa sortie, la patiente a été mise sous corticothérapie orale et inhalée. Après deux semaines, la patiente gardait toujours une toux sèche avec des râles crépitants diffus bilatéraux. La reprise de l´interrogatoire a montré la notion d´un voisin proche éleveur de 200 pigeons voyageurs sur une terrasse. Les sérologies de la maladie du poumon des éleveurs d´oiseaux sont revenues positives; IgG e91 pigeon étaient supérieurs à 200 mgA/L et IgG e218 poulet étaient à 80 mgA/L. Deux mois plus tard, la patiente avait une bonne amélioration clinique sous corticothérapie inhalée associée à une corticothérapie orale et traitement adjuvant et surtout après l´éviction de l´exposition aux oiseaux.

## Discussion

La maladie du poumon des éleveurs d´oiseaux est une entité très rare chez la population pédiatrique, elle représente près des deux tiers des étiologies des pneumopathies d´hypersensibilité [1]. La prévalence des pneumopathies d´hypersensibilité chez l´enfant est estimée à 4 cas par million d´enfants [2]. Cette pathologie est due à une réponse immuno-allergique suite à une exposition aux antigènes aviaires présentes dans les plumes, les excréments, les déjections des oiseaux [3]. Elle survient de manière équivalente dans les deux sexes [1]. Notre patiente a été de sexe féminin. L´âge de survenue est très variable pouvant atteindre le grand enfant mais aussi le nourrisson [4,5]. Nous rejoignons la littérature puisque notre patiente est âgée de 7 ans.

En effet, le mécanisme physiopathologique n´est pas bien élucidé, certains auteurs suggèrent que les antigènes aviaires aboutissent à une dysrégulation immunitaire responsable de formations des IgG [6-8]. D´autres induisent le rôle d´une prédisposition génétique dans la physiopathologie de cette maladie [3]. Les pneumopathies d´hypersensibilité sont généralement classées en trois tableaux différents [9,10]: la forme aigue caractérisée par des symptômes similaires aux bronchopneumopathies virales; des sensations fébriles, céphalées, myalgies, arthralgies et des nausées 2 à 9 heures après contact avec l´antigène. La toux et la dyspnée sont fréquemment observées. Ces signes disparaissent quelques jours spontanément. La forme subaiguë s´observe quelques jours voire quelques semaines après le contact avec les antigènes aviaires. La toux et la dyspnée sont quasi-constantes. Cette forme peut être révélée par une détresse respiratoire et une cyanose nécessitant une hospitalisation.

La forme chronique caractérisée par l´installation sur plusieurs mois d´une dyspnée au repos, une cyanose, une insuffisance respiratoire chronique et une altération de l´état général témoignant l´installation d´une fibrose pulmonaire. L´examen physique révèle le plus souvent des râles crépitants aux deux hémichamps pulmonaires. C´est la forme observée chez notre patiente.

Devant les signes cliniques et radiologiques non spécifiques, d´autres diagnostics différentiels simulent la maladie du poumon des éleveurs d´oiseaux notamment l´asthme, la primo-infection tuberculeuse, les bronchopneumopathies virales. Le diagnostic de cette entité est donc n´est pas facile et reposant sur un faisceau d´arguments anamnestiques, cliniques, radiologiques, biologiques et anatomopathologiques [10]: preuve d´un contact avec les antigènes aviaires: anamnèse et sérologies positives; symptomatologie évocatrice en fonction de la forme (aigue, puis subaiguë puis chronique); imagerie compatible: aspect en verre dépoli, de nombreux micro nodules bilatéraux de l´ensemble du parenchyme pulmonaire, voire un aspect de fibrose pulmonaire dans les stades avancés; alvéolite lymphocytaire au liquide broncho-alvéolaire; spirométrie et transfert du CO dans la limite de la normale.

Dans notre cas, la patiente a été traitée initialement comme asthme devant les épisodes de dyspnée sifflante et le terrain d´atopie familiale chez la mère et le frère, et mise sous traitement de fond sans amélioration satisfaisante, ce qui a retardé le diagnostic. Ce n´est qu´après la reprise de l´interrogatoire que la notion de contact avec les antigènes aviaires est mise en évidence, et confirmée par les sérologies spécifiques. La TDM thoracique est l´examen de choix dans l´évaluation de la maladie du poumon des éleveurs d´oiseaux. Celle-ci peut montrer des opacités en verre dépoli bilatérales dans 75% à 90% des cas, qui prédominent généralement dans les zones périhilaires et basales, associées à des micronodules flous, de type centro lobulaire, qui peuvent être disséminés dans l´ensemble des champs pulmonaires ou un aspect en mosaïque caractéristique [1] ce qui a été le cas de notre patiente. En revanche, la radiographie thoracique a un intérêt plus dans le suivi.

Le traitement repose essentiellement sur l´éviction totale de l´exposition aux antigènes incriminés dans la maladie (plumes, excréments, déjection des oiseaux), associée à une corticothérapie inhalée ou par voie générale dans les formes aigues et subaiguës [6]. Notre patiente avait une forme sévère par conséquent une corticothérapie orale a été instituée. Le pronostic dépend du stade de la maladie, l´amélioration est spectaculaire si le diagnostic est porté précocement ainsi que l´éviction de l´exposition. Une évolution défavorable vers la fibrose et l´insuffisance respiratoire chronique est la conséquence d´un diagnostic tardif et une exposition continue aux antigènes aviaires.

## Conclusion

Malgré que la maladie du poumon des éleveurs d´oiseaux reste une pathologie rare chez l´enfant, cette entité doit être évoquée si les arguments anamnestiques, cliniques, radiologiques, biologiques et anatomopathologiques sont évocateurs. Elle peut être similaire à d´autres pathologies notamment un asthme. Ainsi, tout retard diagnostique peut être responsable d´une évolution défavorable de la maladie voire vers une fibrose pulmonaire.
